# Increased Serum Levels of Mesencephalic Astrocyte-Derived Neurotrophic Factor in Subjects With Parkinson’s Disease

**DOI:** 10.3389/fnins.2019.00929

**Published:** 2019-09-04

**Authors:** Emilia Galli, Anu Planken, Liis Kadastik-Eerme, Mart Saarma, Pille Taba, Päivi Lindholm

**Affiliations:** ^1^Institute of Biotechnology, Helsinki Institute of Life Science (HiLIFE), University of Helsinki, Helsinki, Finland; ^2^North Estonia Medical Centre Foundation, Tallinn, Estonia; ^3^Department of Neurology and Neurosurgery, University of Tartu, Tartu, Estonia; ^4^Department of Neurology, Tartu University Hospital, Tartu, Estonia

**Keywords:** Parkinson’s disease, MANF, CDNF, blood, ELISA

## Abstract

**Background:**

Mesencephalic astrocyte-derived neurotrophic factor (MANF) and cerebral dopamine neurotrophic factor (CDNF) promote the survival of midbrain dopamine neurons in animal models of Parkinson’s disease (PD). However, little is known about endogenous concentrations of MANF and CDNF in human PD patients, and their relation to PD pathogenesis. Our main objective was to study whether circulating concentrations of MANF and CDNF differ between PD patients and controls, and if they correlate with clinical parameters. Levels of circulating CDNF were studied for the first time.

**Methods:**

MANF and CDNF levels were measured from serum samples of 34 PD patients and 35 controls using validated in-lab-designed enzyme-linked immunosorbent assay (ELISAs). *MANF* and *CDNF* mRNA levels in whole blood samples of 60 PD patients and 30 controls were measured by quantitative real time polymerase chain reaction (qRT-PCR). MANF concentrations in different blood cell types were measured by ELISA.

**Results:**

Circulating MANF concentrations were significantly higher in PD patients compared to controls (*P* < 0.001) and were positively correlated with Beck Depression Inventory (BDI) depression rating. MANF protein was present in blood cells, however, *MANF* mRNA levels in the blood did not differ between PD patients and controls (*P* = 0.44). The mean concentration of serum CDNF was 33 pg/ml in the controls. CDNF levels were not altered in PD patients (*P* = 0.25).

**Conclusion:**

MANF but not CDNF level was increased in the blood of PD patients. It would be interesting to examine the blood level of MANF from early stage PD patients in future studies to test whether MANF can be used as a clinical marker of PD.

## Introduction

Parkinson’s disease (PD) is a neurodegenerative disorder leading to severe motor impairment. Loss of midbrain dopamine neurons and accumulation of Lewy bodies are typical pathological features in the brain of PD patients. It has been estimated that by the time PD has been clinically diagnosed, 30% of dopamine neurons in the *Substantia nigra* have been lost (Cheng et al., 2010). Pathological changes are not restricted to the brain, and Lewy bodies are also found in the peripheral nervous system (Del Tredici and Braak, 2016; Poewe et al., 2017). Non-motor symptoms of PD, including sleep disorders, olfactory dysfunction and autonomic dysfunction can be present years before the clinical onset of motor symptoms. Cognitive impairment and depression are also characteristics of PD. Although the current treatment for PD is symptomatic, novel treatments are being investigated for slowing down or reversing the pathogenesis (Poewe et al., 2017).

Mesencephalic astrocyte-derived neurotrophic factor (MANF) and cerebral dopamine neurotrophic factor (CDNF) are endoplasmic reticulum (ER)-localized, secreted proteins with neuroprotective and neurorestorative activities (Lindahl et al., 2017). Originally, MANF was discovered based on its survival-promoting activity on cultured dopamine neurons (Petrova et al., 2003); CDNF was later identified as its paralog (Lindholm et al., 2007). Studies in *Drosophila* and zebrafish indicate that MANF is important for the development of the dopamine system, since its removal decreased dopamine levels and altered the morphology and number of dopamine neurons (Palgi et al., 2009; Chen et al., 2012). The neuroprotective effects of CDNF and MANF have been studied in animal models of PD. For example, it has been shown that delivery of CDNF protein to the brain parenchyma protected dopamine neurons in rat midbrains against 6-hydroxydopamine (6-OHDA) and 1-methyl-4-phenyl-1,2,3,6-tetrahydropyridine (MPTP)-induced degeneration, and promoted functional recovery (Lindholm et al., 2007; Airavaara et al., 2012). The neuroprotective effect of CDNF has also been demonstrated using adeno-associated virus mediated gene delivery in rat 6-OHDA models of PD (Back et al., 2013; Ren et al., 2013). In marmoset monkeys, CDNF promoted dopamine transporter activity in a 6-OHDA model of PD (Garea-Rodriguez et al., 2016). CDNF is currently in phase I-II clinical trials on human PD patients (ClinicalTrials.gov identifier: NCT03295786; Huttunen and Saarma, 2019). Similar to CDNF, MANF has also demonstrated protective effects on midbrain dopamine neurons in rodent 6-OHDA and MPTP models of PD (Voutilainen et al., 2009; Hao et al., 2017; Liu et al., 2018).

Since MANF and CDNF have protective properties for dopamine neurons, they are potential therapeutic proteins for PD. However, less is known about their association with human PD pathophysiology. Here our aim was to study the possible association of circulating MANF and CDNF with human PD pathogenesis. We measured serum MANF and CDNF concentrations from PD patients and controls, and analyzed statistical correlations between their levels and known clinical parameters.

## Materials and Methods

### Study Populations

Two populations were included in the study. Blood samples for enzyme-linked immunosorbent assay (ELISA) analyses were collected from 34 patients with PD and 35 controls (Table 1). PD patients had a mean age of 74.4 ± 8.6 years and controls 75.3 ± 8.2 years. Mean duration of PD was 6.4 ± 6.8 years. Whole blood samples for total RNA isolation and quantitative real time polymerase chain reaction (qRT-PCR) analysis were collected from 60 PD patients and 30 controls (Table 1). The mean age of the PD patients and healthy controls were 73.2 ± 8.0 and 73.0 ± 7.9 years, respectively. The mean duration of PD was 7.1 ± 6.8 years. The study was approved by the Research Ethics Committee of the University of Tartu, and all subjects provided written informed consent.

**TABLE 1:** Demographic and clinical data on the study populations included in ELISA or qRT-PCR analysis.

	**Blood serum/ELISA analysis**		**Whole blood/qRT-PCR analysis**	
**Parameter**	**PD (Mean; range)**	**Control (Mean; range)**	**PD (Mean; range)**	**Control (Mean; range)**
Age (years)	74.4; 48–87	75.3; 58–90	73.2; 48–85	73.0; 58–85
Female:Male	18:16	19:16	34:26	15:15
Duration of PD (years)	6.4; 1–35		7.1; 0.7–35	
MDS-UPDRS (total)	76.7; 26–167		71.3; 22–167	
H&Y	2.9; 1.5–5		2.8; 1–5	
SE-ADL	75.6; 40–100		76.4; 40–100	
MMSE	27; 20–30		27.2; 18–30	
BDI	15.2; 2–32		14.8; 0–32	
SS12	5.7; 0–11		5.8; 0–11	

*BDI, Beck depression inventory; ELISA, enzyme-linked immunosorbent assay; H&Y, Hoehn and Yahr staging; MDS-UPDRS, movement disorder society-unified Parkinson’s disease rating scale; MMSE, mini mental state examination; qRT-PCR, quantitative reverse transcription-polymerase chain reaction; SE-ADL, Schwab–England activities of daily living scale; SS12, sniffing sticks olfactory test.*

### Clinical Assessment

Only patients who fulfilled the Queen Square Brain Bank Criteria for PD (Gibb and Lees, 1988; Poewe et al., 2017) were recruited. Patients with PD underwent medical assessment, which included clinical history, comorbid diseases, toxins in history, and medication use. The disease severity was staged according to the Hoehn and Yahr (H&Y) scale (Hoehn and Yahr, 1967), and the disability was assessed with the Schwab and England Activities of Daily Living (SE-ADL) scale (Schwab et al., 1969). The severity of the PD symptoms was identified by using the Movement Disorders Society-Unified Parkinson’s Disease Rating Scale (MDS-UPDRS), as a rule rated in the ON-stage of the disease (Goetz et al., 2008). Cognitive function was assessed using the Mini Mental State Examination (MMSE) (Folstein et al., 1975). Depression was rated with the Beck Depression Inventory scoring (Beck et al., 1961) based on 21 questions with ratings from 0 to 3 to measure severity of depression. A score of 18 points signifies depression in PD patients (Silberman et al., 2006; Tumas et al., 2008). Olfactory performance was assessed with the Sniffin’ Sticks Olfactory test (SS-12) (Hummel et al., 1997). Current PD medications of the patients involved in the ELISA measurements are provided in Supplementary Table S1. Comorbidities and other physiological or medical conditions of the PD patients are listed in Supplementary Table S2.

### Controls

The control group was formed from healthy volunteers without any current neurological condition, and individuals who visited outpatient or inpatient clinics of the Department of Neurology and Neurosurgery of Tartu University Hospital without any current neurodegenerative disease but because of other neurological conditions.

### Serum Samples

Blood samples were collected to BD Vacutainer^®^ tubes and allowed to clot at room temperature. Serum was separated by centrifugation at 1300 × *g* for 10 min at room temperature (RT), and stored at −80°C until analysis.

### Human MANF ELISA

The development and validation of human MANF ELISA for the measurement of MANF in serum has been described earlier (Galli et al., 2016). The dynamic range of MANF ELISA is 62.5–2000 pg/ml, and its sensitivity is 45 pg/ml. Human MANF ELISA does not recognize recombinant human CDNF. For MANF quantitation, serum samples were diluted at 1:20 in blocking buffer [1% casein in phosphate buffered saline, 0.05% Tween-20 (PBST)]. To block interference caused by heterophilic antibodies (HAs), Immunoglobulin Inhibiting Reagent (IIR; BioIVT) was added to the serum samples at concentration of 500 mg/l. Diluted samples and standards were applied to 96-well plates coated with goat anti-human MANF antibody (AF3748, R&D Systems), and incubated overnight at +4°C in agitation. After washing with PBST, bound MANF on the plates was detected using horseradish peroxidase (HRP)-conjugated mouse anti-human MANF antibody (4E12, Icosagen) and DuoSet ELISA Development System (R&D Systems). Absorbance was read at 450 nm and 540 nm using VICTOR^3^ plate reader.

### Human CDNF ELISA

The development of human pAb CDNF ELISA has been described (Back et al., 2013). Sensitivity of the CDNF ELISA is 6.2 pg/ml (determined as the mean + 3 standard deviations (SDs) of 10 blank samples) and the quantitation range 7.8–500 pg/ml (Supplementary Table S3). The antibodies used in CDNF ELISA were goat anti-hCDNF (1 μg/ml, R&D Systems) for coating, and rabbit anti-CDNF (0.1 μg/ml, Lindholm et al., 2007) followed by HRP-conjugated donkey anti-rabbit (1:2000, GE Healthcare) for detection. The mean intra- and inter-assay variations (analyzed at three different concentration levels) are 9.3 ± 2.5 and 9.7 ± 1.5% CV, respectively. For testing the blockage of HA-interference, we added IIR to serum samples at concentration of 100 and 500 mg/l, and monitored the background with a control ELISA (cELISA) as described (Galli et al., 2016). The cELISA was run exactly as the CDNF ELISA; the only difference was a coating antibody, which was goat anti-hMANF (1 μg/ml, R&D Systems), opposed to goat anti-hCDNF used in the CDNF ELISA. Cross-reactivity of cELISA for neither recombinant CDNF nor MANF was verified at a concentration of 2 ng/ml. Linearity of dilution and recovery of spiked CDNF in serum was tested in the presence of IIR.

### Fractionation of Blood Cells and Quantitation of MANF

For fractionation, we pooled 10 subjects’ blood obtained from the Finnish Red Cross (license 43/2017). A total of 50 ml citrate-blood was centrifuged at 150 × *g* for 20 min at room temperature with no brake applied. The platelet-rich plasma was transferred into a new tube without disturbing the buffy coat, and HEP buffer, pH7.4, (140 mM NaCl, 5 mM EDTA, 3.8 mM HEPES, 2.7 mM KCl) was added at a 1:1 ratio. The sample was mixed gently to prevent platelet activation and centrifuged at 100 × *g* for 15–20 min at RT to pellet contaminating red and white blood cells (RBCs and WBCs). The remaining supernatant was centrifuged at 800 × *g* for 20 min at RT to pellet platelets. Isolated platelets were rinsed twice with 1 mM EDTA in PBS (pH 7.4) and homogenized in 400 μl of lysis buffer (20 mM Tris–HCl, pH 8.2, 137 mM NaCl, 1% NP40, 10% glycerol, 0.5 mM Na_3_VO_4_, Complete Mini protease inhibitor cocktail (Roche)). After 20 min incubation on ice, the sample was centrifuged at 12,000 × *g* for 20 min at + 4°C, and the supernatant was stored at −80°C until analysis.

WBCs were collected by isolating the buffy coat of the initial centrifugation. Contaminating RBCs were lysed by the addition of 0.5 ml of eBioscience^TM^ RBC Lysis Buffer (Invitrogen) and incubated for 15 min at RT. WBCs were pelleted by centrifugation at 500 × *g* for 5 min at RT. The supernatant was decanted, and the pellet was carefully resuspended in cold PBS and centrifuged again. The washed pellet was homogenized in 400 μl of lysis buffer.

For RBC isolation, the top layer contaminated with buffy coats was removed and 400 μl of the RBC layer was collected. 8 ml of PBS was added to the RBC layer and centrifuged at 500 × *g* for 10 min at RT. The supernatant was removed, and the washing was repeated two more times. The pellet of RBCs was homogenized in 800 μl of lysis buffer.

Mesencephalic astrocyte-derived neurotrophic factor in the homogenates was quantitated by hMANF ELISA and the result normalized to the total protein concentration measured by DC Protein Assay (Bio-Rad Laboratories).

### Western Blotting

Protein samples derived from fractionated blood cells were reduced by boiling with β-mercaptoethanol, separated on 4–15% SDS-PAGE (MiniProtean^®^ TGX^TM^, Bio-Rad; 50 μg/lane) and blotted on a nitrocellulose membrane. Proteins were stained with Ponceau S (0.1% in 5% acetic acid) after which MANF was detected with rabbit anti-MANF (1 μg/ml, Icosagen) followed by HRP-conjugated secondary antibody against rabbit IgG and enhanced chemiluminescence (ECL) Western Blotting Substrate kit (Pierce).

### RNA Isolation and Real-Time Quantitative PCR

Whole blood samples were collected into Tempus Blood RNA tubes (Applied Biosystems) and frozen at −80°C. Total RNA was isolated using Tempus Spin RNAS isolation kit (Applied Biosystems). 200 ng of RNA was used for cDNA synthesis using High Capacity cDNA Reverse Transcription Kit with RNase Inhibitor (Applied Biosystems). cDNA was used as a template for TaqMan^®^ qRT-PCR analysis in the ABI Prism 7900HT Sequence Detection System (Applied Biosystems, Foster City, CA, United States). TaqMan^®^ Gene Expression Master Mix and FAM labeled TaqMan (Applied Biosystems) gene assays were used to detect the mRNA expression level of the gene of interest and of actin as a reference gene. The TaqMan probes used were: Hs00180640_m1 (MANF), Hs00418490_m1 (CDNF), and Hs01060665_g1 (ActB). Reactions were carried out in four replicates. Data was analyzed using the 2(-Delta Delta C(T)) method, where the gene expression levels were normalized to the level of the actin beta housekeeping gene.

### Statistical Analysis

The distributions of MANF and CDNF concentrations were skewed (Shapiro–Wilk test, *P* < 0.05 for both), thus non-parametric statistical tests were carried out to analyze serum data. Bivariate correlations were analyzed with Spearman’s correlation test. Kruskal–Wallis, along with Tukey HSD *post hoc* test, and Mann–Whitney *U*-tests were used to compare MANF and CDNF serum levels in different groups. qRT-PCR data was analyzed with the parametric unpaired *t*-test. *P* ≤ 0.05 was considered statistically significant for all tests. Statistical analyses were performed using Software Package for Social Science (SPSS) v. 21.0. All results are expressed as mean ± SD.

## Results

### Serum MANF Levels Are Higher in PD Patients Compared to Controls

**FIGURE 1:**
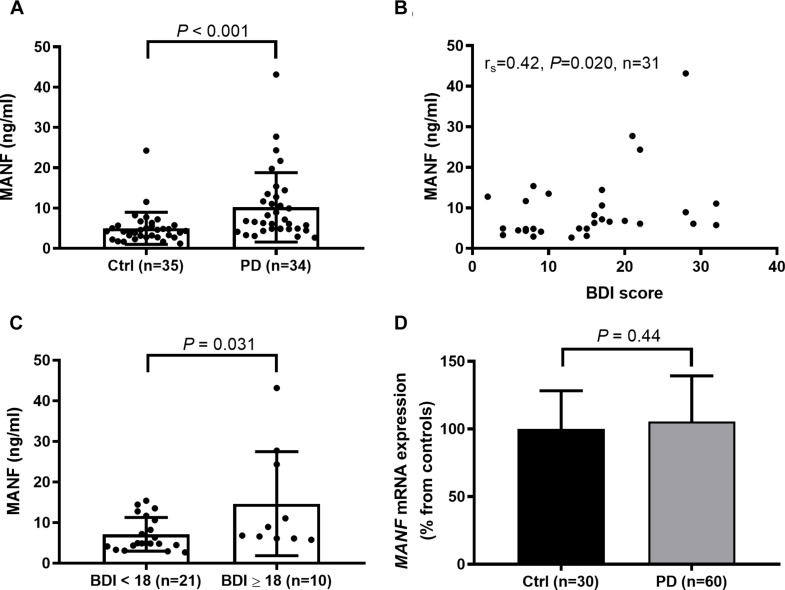
**(A)** Increased levels of circulating MANF (Mann–Whitney *U*-test, *P* < 0.001) in PD patients versus controls as measured by in-lab-designed ELISA. **(B)** Positive correlation between MANF concentration and Beck Depression Inventory (BDI) score in PD patients (*r*_*s*_ = 0.42; *P* = 0.02). **(C)** The average level of serum MANF was higher in PD patients scoring 18 or more BDI points than in patients with scores of 17 or less (Mann–Whitney *U*-test, *P* = 0.031). **(D)** *MANF* mRNA levels do not differ between the blood of PD patients and controls (*t-*test, *P* = 0.44).

The average serum MANF concentration was twice as high in PD patients compared to their age-matched controls (Figure 1A). Serum MANF concentration was 10.2 ± 8.6 ng/ml (median: 6.7 ng/ml, *n* = 34) in PD patients and 5.0 ± 4.0 ng/ml (median: 4.3 ng/ml, *n* = 35) in the control group (*P* < 0.001). The difference in MANF serum concentration between the groups was 5.2 ng/ml, which represent a 105% increase.

### Positive Correlation Between MANF Concentrations and BDI Scoring

Serum MANF level was not dependent on duration or severity of PD (Table 2), or used medication (Supplementary Table S1). However, a positive correlation was found between serum MANF concentration with BDI scoring (*r*_*s*_ = 0.42; *P* = 0.02; Figure 1B). In PD patients who scored 18 or more points the average serum MANF level was 14.7 ± 12.8 ng/ml (*n* = 10), while in patients scoring 2–17 points the average MANF concentration was 7.1 ± 4.2 ng/ml (*n* = 21, *P* = 0.031; Figure 1C).

**TABLE 2:** Correlations between serum MANF and clinical variables in PD patients.

**Variable**	***n***	**Spearman’s Rho**	***P*-value**
Age	34	–0.14	0.45
Duration of PD	34	0.10	0.58
MDS-UPDRS, total score	34	–0.13	0.48
MDS-UPDRS, I	34	0.05	0.79
MDS-UPDRS, II	34	–0.09	0.64
MDS-UPDRS, III	34	–0.21	0.24
MDS-UPDRS, IV	34	0.33	0.06
H&Y	34	–0.07	0.70
SE-ADL	34	0.01	0.94
MMSE	34	0.26	0.15
SS12	32	–0.25	0.17
PDQ39	32	0.25	0.17
BDI	31	0.42	0.02^∗^

*Significant values (*P* ≤ 0.05) are marked with ^∗^. BDI, Beck depression inventory; H&Y, Hoehn and Yahr staging; MDS-UPDRS, movement disorder society-unified Parkinson’s disease rating scale; MMSE, mini mental state examination; SE-ADL, Schwab–England activities of daily living scale; SS12, sniffing sticks olfactory test.*

### MANF mRNA Levels in Blood Cells Do Not Differ Between PD Patients and Controls

It has been reported that *MANF* mRNA is expressed in the peripheral WBCs (Wang J. et al., 2014). Thus, it is possible that circulating MANF is derived from blood cells. We used ELISA to quantitate MANF levels in different types of blood cells isolated from healthy human donors. MANF levels in RBCs, WBCs, and platelets in a pooled blood sample were 0.1, 79.5, and 358.8 ng/mg of total protein, respectively (Supplementary Figure S1A). In agreement, MANF was detected in platelets but not in RBCs by Western blot (Supplementary Figure S1B).

Next, we used qRT-PCR analysis to measure *MANF* mRNA levels in whole blood derived from the PD patients and controls. However, *MANF* transcripts were not elevated in the blood of PD patients compared to controls (*P* = 0.44; Figure 1D).

### CDNF Is Present in Blood Circulation at Low pg/ml-Levels

We have previously found a significant interference in our in-lab-designed hMANF ELISA by HAs present in human blood circulation (Galli et al., 2016). Similar interference was observed in the hCDNF ELISA as analyzed by a cELISA. The cELISA was constructed on an antibody pair derived from the same host species as the antibodies in the original ELISA, but without reactivity for either recombinant CDNF or MANF. Addition of IIR to the serum samples decreased the background detected by the cELISA. The best blockage of background caused by HA was obtained with 1:4 serum dilution and 500 mg/l IIR when analyzed on the hCDNF ELISA (Supplementary Table S4). The recovery of recombinant CDNF added at concentrations of 50, 100, or 250 pg/ml to sera diluted at 1:4 and supplemented with 500 mg/l IIR was 76.6 ± 3.3% (range 72.0–82.8%, *n* = 7). Linearity of dilution of endogenous CDNF in human sera was 95.5 ± 12.1% (*n* = 3 sera in 4 sequential dilutions, Supplementary Table S5).

In general, CDNF levels in serum were much lower than those of MANF. The range of calculated serum CDNF concentrations in the study population was 4.3–134.4 pg/ml. As the lower limit of detection (LLOD) of our hCDNF ELISA is 6.2 pg/ml, and the sera need to be diluted 1:4 for optimal background control, the minimal CDNF concentration that can reliably be distinguished from the blank is 24.8 pg/ml.

### Circulating CDNF Levels Unaltered in PD Patients Compared to Controls

**FIGURE 2:**
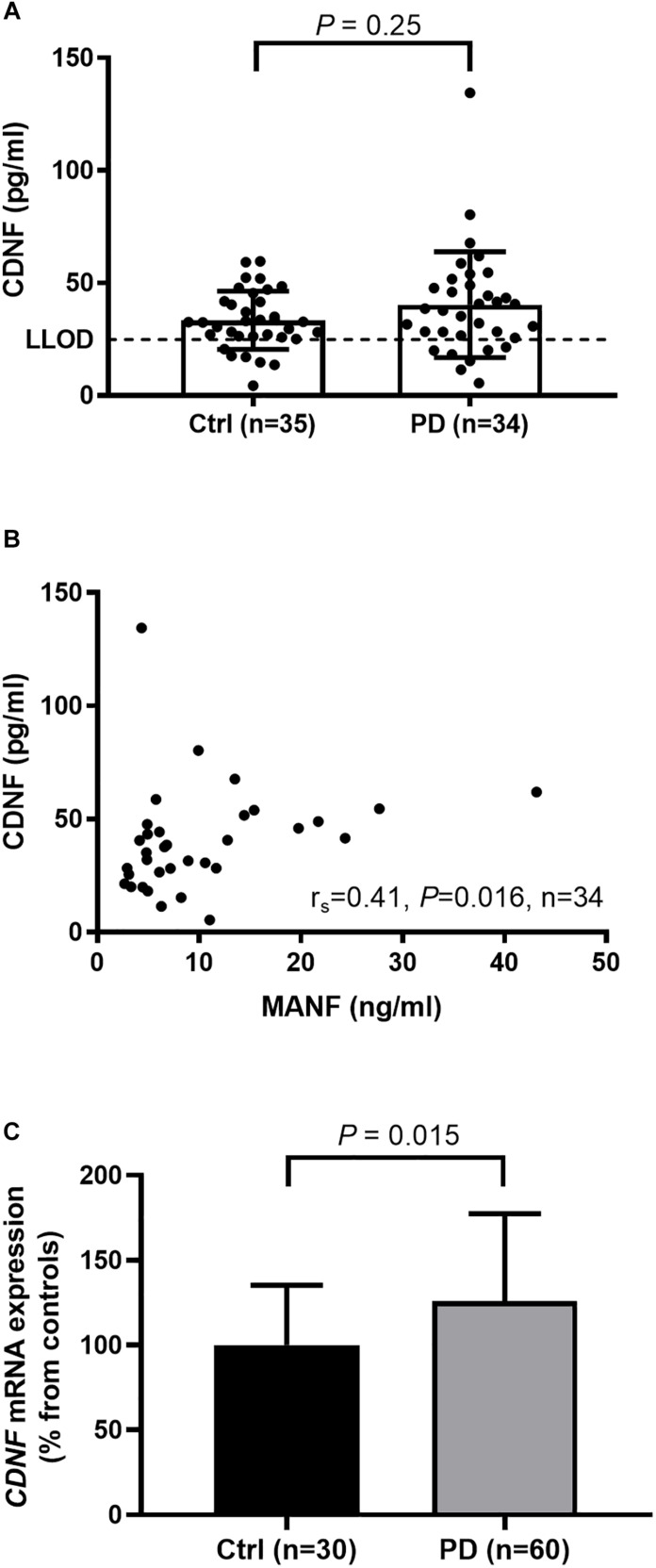
**(A)** Similar levels of serum CDNF in PD patients and controls as measured by in-lab-designed hCDNF ELISA (Mann–Whitney *U*-test, *P* = 0.25). LLOD; lower limit of detection when samples are diluted 1:4. **(B)** Positive correlation of MANF and CDNF levels in the sera of PD patients (*r*_*s*_ = 0.41, *P* = 0.016). **(C)** Increased levels of *CDNF* mRNA in the blood of PD patients compared to controls (*t-*test, *P* = 0.015).

Cerebral dopamine neurotrophic factor (CDNF) concentrations in 7 (21%) samples form the PD and 6 (17%) samples from the control group fell below the LLOD of our hCDNF ELISA. We approached the data analysis by three simple methods used for dealing with samples under LLOD (Helsel, 1990): (1) including all sample values; (2) replacing the sample values below LLOD by 0; and (3) replacing the sample values below LLOD by half of the LLOD (i.e., 12.4 pg/ml), which can be considered as a rough mean value of the samples that fall below the detection limit. All the three methods gave similar results indicating that serum CDNF levels in PD patients and controls do not significantly differ in the current study population. *P*-values for each of the analysis methods were following: (1) 0.25, (2) 0.28, and (3) 0.28. In the Figure 2A we show the values also below the LLOD for not causing an artificial skew in the distribution. However, all values below LLOD should be taken with a level of uncertainty. When all sample values were included in the calculations, the average serum CDNF concentration in the PD patients was 40.3 ± 23.5 pg/ml (median: 38.1 pg/ml, *n* = 34), and in the controls it was 33.3 ± 12.9 pg/ml (median: 32.5 pg/ml, *n* = 35; Figure 2A).

MANF and CDNF concentrations correlated positively in the sera from the PD patients (*r*_*s*_ = 0.41, *P* = 0.016, *n* = 34, Figure 2B) but not in those from the controls (*r*_*s*_ = 0.18, *P* = 0.31, *n* = 35).

### CDNF mRNA Levels Increased in the Blood of PD Patients

Using qRT-PCR we observed that *CDNF* gene expression was upregulated in whole blood samples of PD patients by 26% as compared to controls (*P* = 0.015; Figure 2C).

## Discussion

Since MANF and CDNF promote the survival of brain dopamine neurons in animal models of PD (Lindholm et al., 2007; Voutilainen et al., 2009; Airavaara et al., 2012; Back et al., 2013; Ren et al., 2013; Garea-Rodriguez et al., 2016; Hao et al., 2017; Liu et al., 2018), we wanted to study whether their endogenous levels are altered in human subjects with PD, which would indicate a possible contribution to the disease mechanism. Serum was chosen for the study since its collection is minimally invasive and endogenous levels of circulating MANF and CDNF in human PD have not been addressed before.

The average concentration of circulating MANF was roughly twice as high in the group of PD patients as in the controls. MANF levels did not depend on the duration or progression of PD, or on used medication. However, we found a positive correlation with BDI scoring, which is used to measure severity of depression (Beck et al., 1961). Depression is a common non-motor symptom of PD, which negatively affects the quality of life (Poewe et al., 2017). Depression has been demonstrated as a strongest determinant of low health-related quality of life in persons with PD (Kadastik-Eerme et al., 2015). Compared to patients with lower scores, the average MANF level was significantly higher in patients classified as depressed based on a BDI score of 18 or higher. It is unclear whether the observed increase in MANF is directly related to depression, or whether it reflects PD pathophysiology leading to depression. To this end, it would be informative to analyze MANF levels in non-PD subjects diagnosed with depression, and compare them to healthy subjects and those with PD.

Here, we report for the first time that CDNF can be detected in the blood circulation. The basal levels of CDNF in human circulation are clearly lower than the levels of MANF. The difference between MANF and CDNF levels was approximately 170-fold (i.e., 5 ng/ml vs. 0.03 ng/ml) in the control group. We found a positive correlation between MANF and CDNF levels in the sera of PD patients, suggesting that patients having high MANF serum level also had higher CDNF levels. This correlation was not observed in the control population, implying that the release of MANF and CDNF to the blood circulation might be related to a pathological state. However, unlike MANF, the average levels of CDNF were not altered in subjects with PD, as measured by ELISA. It should be noted that in 19% (13/69) of the samples CDNF-readings remained under detection limit, and thus, a more sensitive ELISA could give a different result to that we report here.

A recent study shows that CDNF levels were increased in the hippocampi of PD patient brain. Differently from CDNF, hippocampal levels of MANF were unaltered in PD (Virachit et al., 2019). Whether circulating and brain tissue levels of CDNF and MANF show any correlations in PD remains to be studied.

Indeed, the origins of MANF and CDNF in serum are still unknown. For example, brain-derived neurotrophic factor is stored in platelets and released upon stimulation (Fujimura et al., 2002). We found that MANF is present in high amounts in the platelets but whether they release MANF into the circulation is unclear. We have not detected differences between average concentrations of MANF in human serum and plasma (unpublished observations), suggesting that clot-forming platelets are not the major source of serum MANF. Furthermore, *MANF* mRNA levels in whole blood did not differ between PD patients and controls, suggesting that the increased MANF in the serum of PD patients was not derived from blood cells. In addition, while we observed increased *CDNF* mRNA levels in the blood of PD patients, circulating levels of CDNF were not significantly changed in PD, suggesting that *CDNF* mRNA levels in blood cells and the levels of circulating CDNF do not correlate.

In the rodent brain, endogenous MANF localizes mainly to neurons, and MANF immunoreactivity has been detected in dopamine neurons (Lindholm et al., 2008; Wang H. et al., 2014). However, tissue expression of MANF is also widespread outside the nervous system (Lindahl et al., 2017). In conditions of disease, it is possible that MANF is secreted or non-specifically released from damaged cells or tissues. Previous studies have shown increased circulating MANF concentrations in prediabetes and type 2 diabetes, and in children with recent onset of type 1 diabetes (Galli et al., 2016; Wu et al., 2017). Since increased serum MANF levels were also observed in PD patients, it is possible that the molecular mechanisms regulating MANF concentrations are similar both in diabetes and in PD.

Detection of the PD pathogenesis before the onset of motor symptoms is an important step toward developing disease-modifying therapies. Our results show that MANF serum concentration was doubled in patients with PD compared to age-matched controls, whereas CDNF level was not increased. In future studies it would be interesting to examine circulating MANF levels from early stage PD patients to test whether MANF can be used as a clinical marker of PD.

## Data Availability

The datasets generated for this study are available on request to the corresponding author.

## Ethics Statement

The study was approved by the Research Ethics Committee of the University of Tartu, and all subjects provided written informed consent.

## Author Contributions

EG, AP, MS, PT, and PL conceived and designed the study. EG, AP, and LK-E acquired the data. EG, AP, PT, and PL analyzed and interpreted the data. EG and PL drafted the manuscript. All authors revised and approved the final manuscript for submission.

## Conflict of Interest Statement

PL and MS are inventors of the CDNF and MANF patents that are owned by Herantis Pharma Plc. MS is also a shareholder of the Herantis Pharma Plc. The remaining authors declare that the research was conducted in the absence of any commercial or financial relationships that could be construed as a potential conflict of interest.
