# Supplementary material for: Increased Serum Levels of Mesencephalic Astrocyte-Derived Neurotrophic Factor in Subjects With Parkinson’s Disease
Source: Front Neurosci. 2019 Sep 4;13:929. doi: 10.3389/fnins.2019.00929 (PMC6737037; doi:10.3389/fnins.2019.00929)
Supplement: Supplementary file 1 [file Data_Sheet_1.PDF]

Supplementary Material

Supplementary Tables

Supplementary Table S1. Analysis of serum MANF concentration in relation to current PD medication (Mann-Whitney U or Kruskall-Wallis test). SD, standard deviation.

Variable	Current treatment	n	MANF (ng/ml ± SD)	*P*-value
Amantadine	yes	3	10.1±3.9	0.38
	no	31	10.2±9.0	
MAO-B inhibitor	yes	8	8.6±6.8	0.60
	no	26	10.7±9.1	
Dopamine agonist	yes	12	12.7±10.8	0.16
	no	22	8.9±7.0	
Levodopa	yes	27	10.3±9.2	0.72
	no	7	10.1±6.5	
Number of medications for PD	1	21	9.2±6.9	0.69
	2	10	12.8±12.5	
	3	3	8.8±1.9	

Supplementary Table S2. Comorbidities and other conditions in the PD patients.

**Condition**	**n**	**%**
Hypertension	20	58.8
Other cardiovascular diseases	10	29.4
Rheumatologic diseases	9	26.5
Currently on medication related to other diseases than PD	30	88.2
Antihypertensive therapy	20	58.8
Current smoker	1	2.9
Smoker in history	6	17.6
Contact with chemical industry (including paints) during lifetime	9	26.5

Supplementary Table S3. Dynamic range of the CDNF ELISA. For each standard point, the mean accuracy and precision values of eight runs are shown. The mean accuracy (relative error; RE) and precision (coefficient of variation; CV) were within 10% RE and 10% CV, respectively. The individual accuracy values were within 10% RE of the nominal values, and precision values were within 15% CV.

**rhCDNF (pg/ml)**	**7.8**	**15.6**	**31.3**	**62.5**	**125.0**	**250.0**	**500.0**
Mean recovery	8.00	15.42	31.19	61.49	124.61	250.44	512.97
Mean % RE	102.4	98.7	99.8	98.4	99.7	100.2	102.6
Mean % CV	5.9	4.8	3.5	2.6	1.9	4.4	4.6

Supplementary Table S4. The effect of serum dilution and immunoglobulin inhibiting reagent (IIR) concentration on the reduction of unspecific background absorbance values from human serum samples in CDNF ELISA. The best blockage of background caused by heterophilic antibodies was obtained with 1:4 serum dilution and 500 mg/l IIR. Abs, absorbance.

	Serum dilution 1:2			Serum dilution 1:4		
	Abs on CDNF ELISA	Abs on control ELISA	Background Abs (% from CDNF ELISA)	Abs on CDNF ELISA	Abs on control ELISA	Background Abs (% from CDNF ELISA)
Serum 1	1.237	1.088	88.0	0.616	0.680	110.4
+ 100 mg/l IIR	0.053	0.015	28.3	0.031	0.010	32.3
+ 500 mg/l IIR	0.046	0.011	23.9	0.019	0.000	0.0
Serum 2	0.026	0.012	46.2	0.012	0.006	50.0
+ 100 mg/l IIR	0.016	0.003	18.8	0.007	0.000	0.0
+ 500 mg/l IIR	0.021	0.007	33.3	0.006	0.000	0.0
Serum 3	0.214	0.167	78.0	0.168	0.140	83.3
+ 100 mg/l IIR	0.026	0.003	11.5	0.013	0.000	0.0
+ 500 mg/l IIR	0.037	0.000	0.0	0.010	0.000	0.0

Supplementary Table S5. Linearity of dilution of endogenous CDNF in human sera as analyzed by CDNF ELISA. Values that fell under assay detection limit (6.2 pg/ml) are shown in blue. The sera were supplemented with IIR. SD, standard deviation.

	Dilution factor	CDNF (pg/ml)	Expected	% From expected
Serum 1	2	41.4	41.4	
	4	20.7	20.7	100.1
	8	10.3	10.4	99.6
	16	3.9	5.2	75.0
Serum 2	2	19.4	19.4	
	4	9.8	9.7	101.2
	8	3.8	4.8	79.0
	16	2.2	2.4	92.7
Serum 3	2	15.7	15.7	
	4	7.6	7.9	96.8
	8	3.9	3.9	100.3
	16	2.3	2.0	115.0
			Average	95.5
			SD	12.1

Supplementary Figure S1.

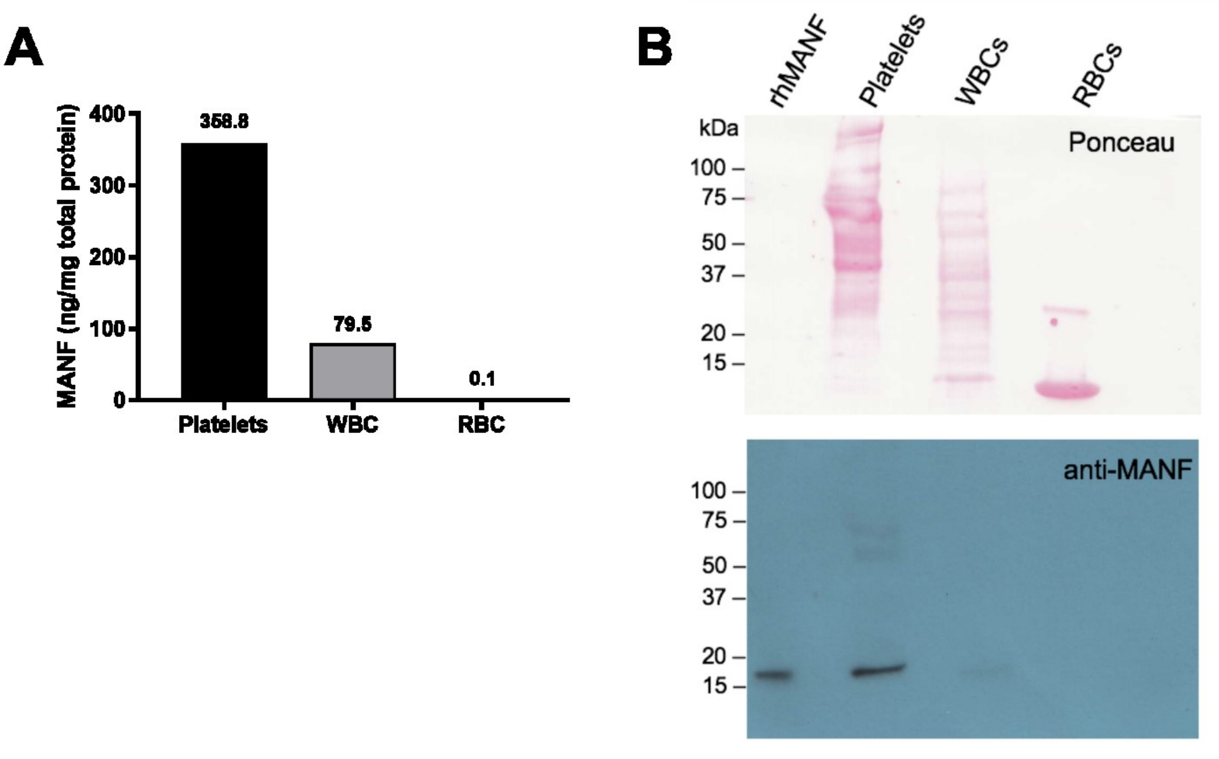

Figure S1. MANF protein is present in the platelets and circulating white blood cells. a) ELISA quantification of MANF levels in blood cell fractions. b) SDS-PAGE analysis of MANF in blood cell fractions. Upper panel: Ponceau-staining of the proteins, loaded as 50 µg of total protein/lane. Lower panel: anti-MANF staining of recombinant human MANF (rhMANF, 10 ng), and MANF in platelets (18 ng/50 µg total protein, based on ELISA measurements), white blood cells (WBCs, 4 ng/50 µg), and red blood cells (RBCs, 0.1 ng/50 µg).
